# Supplementary material for: Joint reconstruction-segmentation on graphs
Source: SIAM J Imaging Sci. Author manuscript; Available in PMC 2026 Nov 5. (PMC7619706; doi:10.1137/22m151546x)
Supplement: Supplementary file 1 [file EMS218490-supplement-Appendix.pdf]

A Computing g_n

In this appendix we explain how we compute g_n, which is defined in Section 4. We recall from Section 3.1 that $z := \mathcal{F}(x)$, and likewise we define $z_n := \mathcal{F}(x_n)$. Then

$$g_n = \mathcal{F}^*(\nabla_z \mathscr{F}_1(z_n) + \nabla_z \mathscr{F}_2(z_n)).$$

We now compute each term in turn. To compute $\nabla_z \mathscr{F}_1(z)$, note that

$$\begin{aligned}\mathscr{F}_1(z+\delta z) &= \beta\langle (G_n)_{YY}, \Omega_{YY}(z+\delta z)\rangle_F \\ &= \beta\left\langle (G_n)_{YY}, \Omega_{YY}(z) + [\langle \nabla_z \Omega_{ij}(z), \delta z\rangle_F]_{i,j\in Y}\right\rangle_F + o(\delta z) \\ &= \mathscr{F}_1(z) + \beta \sum_{i,j\in Y} (G_n)_{ij} \sum_{l\in Y}\sum_{r=1}^{q} (\nabla_z \Omega_{ij}(z))_{lr}\delta z_{lr} + o(\delta z)\end{aligned}$$

and thus for all $l \in Y$ and $r \in \{1, ..., q\}$

$$(\nabla_z \mathscr{F}_1(z))_{lr} = \beta \sum_{i,j\in Y} (G_n)_{ij} (\nabla_z \Omega_{ij}(z))_{lr}.$$

Now, since $(\Omega_{YY})_{ij}(z) = e^{-\|z_i - z_j\|_2^2/q\sigma^2}$ for $i \neq j$ and $(\Omega_{YY})_{ii}(z) = 0$, we have

$$\nabla_{z_{lr}}(\Omega_{YY})_{ij}(z) = \frac{2}{q\sigma^2}\begin{Bmatrix} 0, & l \notin \{i,j\} \\ (\Omega_{YY})_{il}(z)(z_{ir} - z_{lr}), & j = l \\ (\Omega_{YY})_{lj}(z)(z_{lr} - z_{jr}), & i = l \end{Bmatrix} = \frac{2}{q\sigma^2}(\Omega_{YY})_{ij}(z)(z_{ir} - z_{jr})(\delta_{jl} - \delta_{il}),$$

where δ denotes the Kronecker delta. Therefore,

$$(\nabla_z \mathscr{F}_1(z))_{lr} = \frac{2\beta}{q\sigma^2} \sum_{i,j\in Y} (G_n)_{ij}\Omega_{ij}(z)(z_{ir} - z_{jr})(\delta_{jl} - \delta_{il}).$$

Hence, letting $\mathcal{A}(z) := (G_n)_{YY} \odot \Omega_{YY}(z)$ (where $\odot$ denotes the Hadamard product),

$$(\nabla_z \mathscr{F}_1(z))_{lr} = \frac{2\beta}{q\sigma^2} \sum_{i,j\in Y} \mathcal{A}_{ij}(z)(z_{ir} - z_{jr})(\delta_{jl} - \delta_{il}) = \frac{4\beta}{q\sigma^2}\left(\sum_{j\in Y} \mathcal{A}_{lj}(z) z_{jr} - z_{lr}\sum_{j\in Y}\mathcal{A}_{lj}(z)\right)$$

since $\mathcal{A}(z)$ is symmetric, and therefore

$$\nabla_z \mathscr{F}_1(z) = \frac{4\beta}{q\sigma^2}\left(\mathcal{A}(z)z - (\mathcal{A}(z)\mathbf{1}_N)\odot z\right).$$

To compute $\nabla_z \mathscr{F}_2(z)$, note that by a similar argument as the above,

$$(\nabla_z \mathscr{F}_2(z))_{lr} = -\frac{4\beta}{q\sigma^2} \sum_{i\in Y, j\in Z} (G_n)_{ij}(\Omega_{YZ})_{ij}(z, z_d)(z_{ir} - (z_d)_{jr})\delta_{il},$$

for all $l \in Y$ and $r \in \{1, ..., q\}$. Hence, letting $\mathcal{B}(z) := (G_n)_{YZ} \odot \Omega_{YZ}(z, z_d)$, we get

$$(\nabla_z \mathscr{F}_2(z))_{lr} = \frac{4\beta}{q\sigma^2} \sum_{i\in Y, j\in Z} \mathcal{B}_{ij}(z)((z_d)_{jr} - z_{ir})\delta_{il} = \frac{4\beta}{q\sigma^2}\left(\sum_{j\in Z}\mathcal{B}_{lj}(z)(z_d)_{jr} - z_{lr}\sum_{j\in Z}\mathcal{B}_{lj}(z)\right)$$

and therefore we arrive at a similar formula as for $\nabla_z \mathscr{F}_1(z)$:

$$\nabla_z \mathscr{F}_2(z) = \frac{4\beta}{q\sigma^2}\left(\mathcal{B}(z)z_d - (\mathcal{B}(z)\mathbf{1}_{N_d})\odot z\right).$$

Tying this all together, we get

$$\begin{aligned} g_n &= \frac{4\beta}{q\sigma^2}\mathcal{F}^*\big(\mathcal{A}(z_n)z_n + \mathcal{B}(z_n)z_d - (\mathcal{A}(z_n)\mathbf{1}_N + \mathcal{B}(z_n)\mathbf{1}_{N_d})\odot z_n\big) \\ &= \frac{4\beta}{q\sigma^2}\mathcal{F}^*\left(\mathcal{C}_n\begin{pmatrix} z_n \\ z_d\end{pmatrix} - (\mathcal{C}_n \mathbf{1}_{N+N_d})\odot z_n\right),\end{aligned} \tag{A.1}$$

where $\mathcal{C}_n := (G_n)_{YV} \odot \Omega_{YV}(z_n, z_d)$. To compute (A.1), we need to compute matrix-vector products of the form $\mathcal{C}_n v$. Recalling (4.1), it follows that

$$(G_n)_{YV} = -u_n|_Y u_n^T + v_n|_Y \mathbf{1}_V^T + \mathbf{1}_Y v_n^T.$$

Next, we observe a neat linear algebra result[12]

$$\left((-u_n|_Y u_n^T + v_n|_Y \mathbf{1}_V^T + \mathbf{1}_Y v_n^T) \odot A\right) v = -u_n|_Y \odot (A(u_n \odot v)) + v_n|_Y \odot (Av) + A(v_n \odot v),$$

where in this case $A = \Omega_{YV}(z_n, z_d)$. Hence it suffices to be able to compute terms of the form $\Omega_{YV}(z_n, z_d)v$. Via the Nyström extension (2.1) we have

$$\Omega_{YV}(\mathcal{F}(x_n), z_d)v \approx \left(\Omega_{VX}(\mathcal{F}(x_n), z_d)\Omega_{XX}^{-1}(\mathcal{F}(x_n), z_d)\Omega_{XV}(\mathcal{F}(x_n), z_d)v\right)|_Y, \tag{A.2}$$

where $X \subseteq V$ is some interpolation set, so we can compute such products quickly.

These considerations lead us to Algorithm 4 to compute $\mathcal{C}_n v$ for $v \in \mathcal{V}$.

Algorithm 4 Definition of the `CProd` function to be used in Algorithm 1.

function CProd$(z_n, v, u, v_n, \sigma, V, Y, Z, K)$ ▷ Approximates $\mathcal{C}_n v$ as above
    $B : w \mapsto (\texttt{OmegaProd}(w, z_n, z_d, q, \sigma, V, Y, Z, K))|_Y$ ▷ See below
    return $-u|_Y \odot B(u \odot v) + v_n|_Y \odot B(v) + B(v_n \odot v)$
end function
function OmegaProd$(v, z, z_d, q, \sigma, V, Y, Z, K)$ ▷ Approximates $\Omega(z, z_d)v$ via the Nyström extension as in (A.2)
    $\omega : ij \mapsto \Omega_{ij}(z, z_d, q, \sigma)$ ▷ Defined as in (3.1)
    $X = \texttt{random\_subset}(Y, K/2) \cup \texttt{random\_subset}(Z, K/2)$
    $(\omega_{XX}, \omega_{VX}) = (\omega(X, X), \omega(V, X))$
    $\omega_{XX} v' = \omega_{VX}^T v$ ▷ Solving the linear system for v'
    return $\omega_{VX} v'$
end function

B Primal-dual optimisation methods for (4.3)

To solve (4.3), we shall be employing an algorithm of Chambolle and Pock [16]. Following [16], we rewrite (4.3) as:

$$\min_{x \in \mathbb{R}^{N \times \ell}} R(\mathcal{K}x) + G(x), \tag{B.1}$$

where $G(x) := \alpha\|\mathcal{T}(x) - y\|_F^2 + \eta_n\|x - \tilde{x}_n\|_F^2$. Then the primal problem (B.1) can be reformulated as the primal-dual saddle point problem (we recall Footnote 3 for the definition of R^*)

$$\min_{x \in \mathbb{R}^{N \times \ell}} \max_{p \in \mathcal{K}(\mathbb{R}^{N \times \ell})} \langle \mathcal{K}x, p \rangle + G(x) - R^*(p). \tag{B.2}$$

Reformulating (B.1) as (B.2) suggests approaching the minimiser of (B.1) by alternately updating a sequence of x_n and p_n. The algorithm we shall be using, i.e. [16, Algorithm 2], is a sophistication of this basic idea, and is summarised as Algorithm 5. For details and a convergence analysis, see [16]. We shall assume that for any $\delta t > 0$ we can efficiently compute the proximal operator of $\delta t R^*$, $\operatorname{prox}_{\delta t R^*}$.[13] So to employ Algorithm 5,

[12] To see this, observe that in suffix notation, for $i \in Y$ and $j \in V$, the LHS is $(-(u_n)_i(u_n)_j + (v_n)_i + (v_n)_j)A_{ij}v_j$ and the RHS is $-(u_n)_i A_{ij}((u_n)_j v_j) + (v_n)_i A_{ij} v_j + A_{ij}((v_n)_j v_j)$.

[13] For a proper, l.s.c., and convex function f, the *proximal (a.k.a. resolvent) operator of* f is defined by $\operatorname{prox}_f(x') := \operatorname{argmin}_x f(x) + \frac{1}{2}\|x - x'\|^2$. As mentioned in [16], Moreau's identity allows $\operatorname{prox}_{\delta t R^*}$ to be computed via $\operatorname{prox}_{\delta t^{-1} R}$. Since computing $\operatorname{prox}_{\delta t^{-1} R}$ is equivalent to performing a denoising regularised by R, this is a place where "Plug-and-Play" denoising methods (see Venkatakrishnan *et al.* [50]) could be employed instead of the explicit regularisation methods we will use in this paper.

Algorithm 5 Algorithm for solving (B.1), using [16, Algorithm 2].

function PrimalDual($x_0, \gamma, \mathcal{K}, \mathcal{K}^*, \texttt{proxRS}, \texttt{proxG}$) ▷ γ must satisfy [16, (35)]
  $p_0 = \mathcal{K}(x_0)$
  $\bar{x}_0 = x_0$
  $(\delta t_{0,1}, \delta t_{0,2}) = (1/\|\mathcal{K}\|, 0.99/\|\mathcal{K}\|)$ ▷ We must have $\delta t_{0,1}\delta t_{0,2}\|\mathcal{K}\|^2 \leq 1$
  $n = 0$
  while stopping condition not met **do**
    $p_{n+1} = \texttt{proxRS}(p_n + \delta t_{n,1}\mathcal{K}(\bar{x}_n), \delta t_{n,1})$ ▷ $\texttt{proxRS}(p, \delta t) := \text{prox}_{\delta t R^*}(p)$
    $x_{n+1} = \texttt{proxG}(x_n - \delta t_{n,2}\mathcal{K}^*(p_{n+1}), \delta t_{n,2})$ ▷ $\texttt{proxG}(x, \delta t) := \text{prox}_{\delta t G}(x)$
    $\theta_n = (1 + 2\gamma\delta t_{n,2})^{-\frac{1}{2}}$
    $(\delta t_{n+1,1}, \delta t_{n+1,2}) = (\delta t_{n,1}\theta_n^{-1}, \delta t_{n,2}\theta_n)$
    $\bar{x}_{n+1} = x_{n+1} + \theta_n(x_{n+1} - x_n)$
    $n = n + 1$
  end while
  return x_n
end function

we need two ingredients: a method for computing $\text{prox}_{\delta t G}$, and a γ obeying [16, (35)], i.e. such that for all $x, x' \in \mathbb{R}^{N\times\ell}$,

$$G(x') \geq G(x) + \langle \nabla_x G(x), x' - x\rangle_F + \frac{1}{2}\gamma\|x - x'\|_F^2.$$

First, assume that $\mathcal{T}$ is linear. Then it follows that $\nabla_x G(x) = 2\alpha\mathcal{T}^*(\mathcal{T}(x) - y) + 2\eta_n(x - \tilde{x}_n)$ and thus we require that

$$\begin{aligned}
\frac{1}{2}\gamma\|x - x'\|_F^2 &\leq \alpha\langle\mathcal{T}(x) + \mathcal{T}(x') - 2y, \mathcal{T}(x') - \mathcal{T}(x)\rangle_F + \eta_n\langle x + x' - 2\tilde{x}_n, x' - x\rangle_F \\
&\quad - \langle 2\alpha\mathcal{T}^*(\mathcal{T}(x) - y) + 2\eta_n(x - \tilde{x}_n), x' - x\rangle_F \\
&= \alpha\left(\langle\mathcal{T}(x) + \mathcal{T}(x') - 2y, \mathcal{T}(x' - x)\rangle_F - 2\langle\mathcal{T}(x) - y, \mathcal{T}(x' - x)\rangle_F\right) + \eta_n\|x - x'\|_F^2 \\
&= \alpha\|\mathcal{T}(x' - x)\|_F^2 + \eta_n\|x - x'\|_F^2,
\end{aligned}$$

and hence it suffices to take $\gamma = 2\eta_n$. Finally, recall that $\text{prox}_{\delta t G}$ is defined by

$$\text{prox}_{\delta t G}(x) = \operatorname*{argmin}_{x' \in \mathbb{R}^{N\times\ell}} G(x') + \frac{\|x' - x\|_F^2}{2\delta t}$$

which has unique minimiser x' solving $\nabla_x G(x') + \frac{1}{\delta t}(x' - x) = 0$. Solving for x', we get:

$$\text{prox}_{\delta t G}(x) = \left((\delta t^{-1} + 2\eta_n)I + 2\alpha\mathcal{T}^*\mathcal{T}\right)^{-1}\left(2\alpha\mathcal{T}^*(y) + 2\eta_n\tilde{x}_n + \frac{x}{\delta t}\right). \tag{B.3}$$

If $\mathcal{T}$ is non-linear, things are more difficult. There may not exist a valid γ, and if so one must use a method such as [16, Algorithm 1] to solve (4.3), which has slower convergence. We must still compute $\text{prox}_{\delta t G}(x)$. Since $\mathcal{T}$ is assumed to be differentiable, for all x there exists a linear map $D\mathcal{T}_x$ such that $\mathcal{T}(x + \delta x) = \mathcal{T}(x) + D\mathcal{T}_x(\delta x) + o(\delta x)$. Then it follows that

$$\nabla_x G(x) = 2\alpha(D\mathcal{T}_x)^*(\mathcal{T}(x) - y) + 2\eta_n(x - \tilde{x}_n)$$

and hence $x' := \text{prox}_{\delta t G}(x)$ solves

$$(1 + 2\delta t\eta_n)x' + 2\alpha\delta t(D\mathcal{T}_{x'})^*(\mathcal{T}(x') - y) = x + 2\delta t\eta_n\tilde{x}_n. \tag{B.4}$$

Finally, (B.4) can be solved by numerical root-finding methods (see e.g. [41, §9]).

C Computing the SDIE scheme

In this appendix we describe how we compute the SDIE scheme that is described in Section 5.1. By Theorem 5.2, an SDIE update has two steps: a fidelity-forced diffusion and a piecewise linear thresholding. The thresholding is trivial to compute, but the diffusion is non-trivial. Our method for computing fidelity-forced diffusion was described in detail in [11, §5.2.6], so here we only reproduce the key details.

By Theorem 5.1, given u_n and the parameter $\tau > 0$, we seek to compute

$$\mathcal{S}_\tau u_n = e^{-\tau(\Delta+M')}u_n + b,$$

where $b := F_\tau(\Delta + M')M'f'$. To compute $e^{-\tau(\Delta+M')}u_n$, we use the Strang formula [45]:

$$e^{-\tau(\Delta+M')} = \left(e^{-\frac{\tau}{2m}M'}e^{-\frac{\tau}{m}\Delta}e^{-\frac{\tau}{2m}M'}\right)^m + \mathcal{O}\left(m^{-2}\right) = \left(e^{-\frac{1}{2}\delta t M'}e^{-\delta t\Delta}e^{-\frac{1}{2}\delta t M'}\right)^m + \mathcal{O}\left(\delta t^2\right),$$

where $m \in \mathbb{N}$ and $\delta t := \tau/m$. Computing matrix-vector products with $e^{-\frac{1}{2}\delta t M'}$ is straightforward, since M' is a diagonal matrix. To compute matrix-vector products with $e^{-\delta t\Delta}$, we will compute an approximate eigendecomposition of Δ using the Nyström-QR method (recommended by Alfke *et al.* [3]) described in Algorithm 6. For details on this method, see [11, §5.2.3].

Note C.1. *Algorithm 6 really computes an approximate decomposition $U_s\Sigma U_s^T$ of $\tilde{\omega} := D^{-1/2}\omega D^{-1/2}$, and then makes a further approximation $\Delta_s = I - \tilde{\omega} \approx U_s(I_K - \Sigma)U_s^T = U_s\Lambda U_s^T$, where I_K is the $K \times K$ identity matrix, and so on for the random walk Laplacian.*

Algorithm 6 Nyström-QR method for computing an approximate SVD of Δ or Δ_s.

function NYSTRÖMQR($ij \mapsto \omega_{ij}, V, Z, K_1, K_2$) ▷ Computes U_1, Λ, and U_2, where $\Delta \approx U_1\Lambda U_2^T$ is an approximate SVD of rank $K := K_1 + K_2$
  $X = \texttt{random\_subset}(V \setminus Z, K_1) \cup \texttt{random\_subset}(Z, K_2)$
  $(\omega_{XX}, \omega_{VX}) = (\omega(X,X), \omega(V,X))$
  $\hat{d} = \omega_{VX}\left(\omega_{XX}^{-1}\left(\omega_{VX}^T\mathbf{1}\right)\right)$ ▷ Uses (2.1) to approximate $d = \omega\mathbf{1}$
  $\tilde{\omega}_{VX} = \hat{d}^{-1/2} \odot \omega_{VX}$ ▷ Applying $\odot$ columnwise, i.e. $(\tilde{\omega}_{VX})_{ij} = \hat{d}_i^{-1/2}(\omega_{VX})_{ij}$
  $[Q, R] = \texttt{thin\_qr}(\tilde{\omega}_{VX})$ ▷ Computes thin QR decomposition $\tilde{\omega}_{VX} = QR$
  $S = R\omega_{XX}^{-1}R^T$ ▷ N.B. $S \in \mathbb{R}^{K\times K}$
  $S = (S + S^T)/2$ ▷ Corrects symmetry-breaking computational errors
  $[\Phi, \Sigma] = \texttt{eig}(S)$ ▷ Computes eigendecomposition $S = \Phi\Sigma\Phi^T$
  $\Lambda = I_K - \Sigma$
  $U_s = Q\Phi$ ▷ $\Delta_s \approx U_s\Lambda U_s^T$, so to return the decomposition of Δ_s terminate here
  $U_1 = \hat{d}^{-1/2} \odot U_s$ ▷ I.e. $(U_1)_{ij} = \hat{d}_i^{-1/2}(U_s)_{ij}$
  $U_2 = \hat{d}^{1/2} \odot U_s$ ▷ I.e. $(U_2)_{ij} = \hat{d}_i^{1/2}(U_s)_{ij}$
  return U_1, Λ, U_2
end function

In [11,13], this Nyström-QR approximate decomposition was used to approximate the matrix exponential via $e^{-\delta t\Delta} \approx I + U_1(e^{-\delta t\Lambda} - I_K)U_2^T$. But by Note C.1, $\Delta \approx I - U_1\Sigma U_2^T$ is a slightly more accurate approximation, and so we have the improved approximation:

$$e^{-\delta t\Delta} \approx e^{-\delta t}\left(I + U_1(e^{\delta t\Sigma} - I_K)U_2^T\right).$$

Therefore, we compute $v_m \approx e^{-\tau(\Delta+M')}u_n$ by defining $v_0 := u_n$, and v_r for $r \in \{1, ..., m\}$ by

$$\begin{aligned} v_{r+1} &= e^{-\delta t}e^{-\delta t M'}v_r + e^{-\delta t}e^{-\frac{1}{2}\delta t M'}U_1(e^{\delta t\Sigma} - I_K)U_2^Te^{-\frac{1}{2}\delta t M'}v_r \\ &= a_1(\delta t) \odot v_r + a_3(\delta t) \odot \left(U_1\left(a_2(\delta t) \odot \left(U_2^T\left(a_3(\delta t) \odot v_r\right)\right)\right)\right) \end{aligned} \tag{C.1}$$

where $a_1(\delta t) := \exp(-\delta t(\mu' + \mathbf{1}))$, $a_2(\delta t) := \exp(\delta t\,\mathrm{diag}(\Sigma)) - \mathbf{1}_K$, and $a_3(\delta t)$ is the elementwise square root of $a_1(\delta t)$ (where exp is applied elementwise, and $\mathbf{1}_K$ is the vector of K ones).

Finally we note that, by Theorem 5.1, b is the fidelity-forced diffusion with initial condition $u_0 = \mathbf{0}$ at time τ. We compute b via the semi-implicit Euler scheme used for fidelity-forced diffusion in [34]. To compute this, again we use the Nyström-QR decomposition of Δ.

Note C.2. *We do not use the scheme from [34] for all of the fidelity-forced diffusions because the Strang formula is more accurate for the* $e^{-\tau(\Delta+M')}u_n$ *term, see [11, §5.2.5–5.2.7] for details.*

D Examples of sub-analytic regularisers

The following theorem shows that the examples of regularisers $\mathcal{R}$ that are given in Note 7.6, do indeed satisfy the conditions required by Assumption 7.5.

Theorem D.1. *The following functions are semi-analytic, bounded below, and continuous on their domain (which is* $\mathbb{R}^n$*):*

i. $f : x \mapsto \|Ax\|_1$ *for* $A \in \mathbb{R}^{m\times n}$.

ii. A feedforward neural network $\mathcal{NN} := f_L \circ \cdots \circ f_1$ *where* $f_j(x') := \rho(A^{(j)}x' + b^{(j)})$, $A^{(j)} \in \mathbb{R}^{n_j\times n_{j-1}}$, $b^{(j)} \in \mathbb{R}^{n_j}$, $n_0 = n$, $n_L = 1$, *and* $(\rho(x'))_i := \max\{0, x'_i\}$ *(the ReLU function).*

Proof. i. $\operatorname{Gr} f = \{(x, \|Ax\|_1) \mid x \in \mathbb{R}^n\}$ can be written as

$$\bigcup_{e\in\{-1,1\}^m} \bigcap_{j=1}^{m} \left\{ (z_1, z_2) \in \mathbb{R}^{n+1} \;\middle|\; z_2 - \sum_{i=1}^{m} e_i(Az_1)_i = 0 \text{ and } e_j(Az_1)_j \geq 0 \right\}$$

and thus $\operatorname{Gr} f$ is a semi-analytic set. The other properties are trivial.

ii. $\mathcal{NN}$ is a composition of piecewise linear continuous functions, and is hence piecewise linear and continuous. It follows that it is semi-analytic. It is bounded below due to ρ applying the ReLU function. □
